# OpenEP: an open-source simulator for electroporation-based tumor treatments

**DOI:** 10.1038/s41598-020-79858-y

**Published:** 2021-01-14

**Authors:** Matías Marino, Emmanuel Luján, Esteban Mocskos, Guillermo Marshall

**Affiliations:** 1 Laboratorio de Sistemas Complejos, Departamento de Computación and Instituto de Física del Plasma, Facultad de Ciencias Exactas y Naturales-CONICET, C1428EGA CABA, Argentina; 2 Departamento de Computación, Facultad de Ciencias Exactas y Naturales, Universidad de Buenos Aires, C1428EGA CABA, Argentina; 3 Laboratorio Interdisciplinario de Computación de Alto Rendimiento, Departamento de Computación, Facultad de Ciencias Exactas y Naturales, Universidad de Buenos Aires, C1428EGA CABA, Argentina; 4 Centro de Simulación Computacional para Aplicaciones Tecnológicas, CONICET, C1425FQD CABA, Argentina

**Keywords:** Cancer therapy, Computational models, Computational platforms and environments, Software

## Abstract

Electroporation (EP), the increase of cell membrane permeability due to the application of electric pulses, is a universal phenomenon with a broad range of applications. In medicine, some of the foremost EP-based tumor treatments are electrochemotherapy (ECT), irreversible electroporation, and gene electrotransfer (GET). The *electroporation* phenomenon is explained as the formation of cell membrane pores when a transmembrane cell voltage reaches a threshold value. Predicting the outcome of an EP-based tumor treatment consists of finding the electric field distribution with an electric threshold value covering the tumor (electroporated tissue). Threshold and electroporated tissue are also a function of the number of pulses, constituting a complex phenomenon requiring mathematical modeling. We present OpenEP, an open-source specific purpose simulator for EP-based tumor treatments, modeling among other variables, threshold, and electroporated tissue variations in time. Distributed under a free/libre user license, OpenEP allows the customization of tissue type; electrode geometry and material; pulse type, intensity, length, and frequency. OpenEP facilitates the prediction of an optimal EP-based protocol, such as ECT or GET, defined as the critical pulse dosage yielding maximum electroporated tissue with minimal damage. OpenEP displays a highly efficient shared memory implementation by taking advantage of parallel resources; this permits a rapid prediction of optimal EP-based treatment efficiency by pulse number tuning.

## Introduction

The application of short electric pulses with sufficient intensity to biological tissues can increase the cell membrane permeability. This technique, referred to as *electropermeabilization* (EP), encompasses several biophysical and biochemical mechanisms, particularly, the formation of aqueous pores in the cell membrane, also known as *electroporation*^1^. At the tissue scale, it induces several changes such as the electrical conductivity, temperature, and pH, and it can even damage certain tissue areas^2–6^. Depending on the pulse amplitudes and duration the permeabilization or electroporation can be reversible or irreversible. Presently, EP is being applied in a wide range of scientific and industrial areas^6^, such as in medicine, biotechnology, food processing, and environmental preservation, among others. In medicine, there are several EP-based treatments for tumor ablation or DNA delivery, the most important being Electrochemotherapty (ECT)^7^, Irreversible Electroporation (IRE)^8^ and Gene Electro-Transfer (GET)^9^. With the aim of understanding and optimizing EP-based treatments in terms of electrical variables, several parameters must be considered: pulse duration, frequency, number of pulses, applied voltage, number of electrodes, and their placement, among others. In this context, mathematical and computational modeling became a powerful tool for studying and predicting the outcome of EP-based protocols.

The *electroporation* phenomenon is explained as the formation of aqueous pores in the cell membrane when a transmembrane voltage induced by a given pulsing protocol reaches a threshold value. This is known as the *standard* EP * model* or phenomenological model. A natural extension to tissue electroporation dictates that any region of the tissue is electroporated when the electric field induced by a given pulsing protocol reaches a threshold value.

Thus, predicting the outcome of EP-based treatments in terms of electrical variables consists of computing the electric field distribution due to the pulsing protocol and finding the appropriate extent of the electroporated tissue with an associated threshold. Assuming a stationary process and the validity of Ohm’s law, the electric field distribution can be obtained from the solution of the nonlinear Laplace equation for the electrostatic potential (with a nonlinear tissue electric conductivity made a function of the electric field and the temperature through the Penne’s Bioheat equation), and the threshold from experimental measurements. This can be named the *standard mathematical-computational* EP * model* or more concisely the *standard computational* EP * model*. Another version of this model consists of measuring experimentally the electroporated area and choosing the threshold as the electric field isoline that matches the electroporated area.

Looking for optimal EP-based treatment in terms of pulse number, the electroporated tissue, and threshold variations in time enter into the picture. The *standard computational* EP * model* used for this purpose is time-invariant and must be extended to account for it. The analysis of many experimental results from the literature made in Lujan et al.^2^ showed that, for a given range of electric parameters, the electroporated tissue increases logarithmically in time while the threshold decays and the electric field remains almost constant. Assuming this variation in time as a succession of steady states, the *standard computational* EP * model* can be replicated for n consecutive pulses, via the experimental measurement in time of the successive thresholds. Because experimental data of this threshold variation in time is lacking, based on the results from the literature previously discussed, an exponential time decay function for the threshold was proposed in Lujan et al.^2^. This is concisely named the *extended standard computational* EP * model*. The exponential time decay function of the threshold was experimentally corroborated in Marino et al.^10^.

Damage induced by pH changes, thermal damage, and damage due to irreversible EP (in the case of ECT and GET treatments) were not considered in previous discussions of EP-based treatments. Damage due to pH was firstly studied in Electrolyte Ablation (EA), another non-thermal ablative method consisting in the application of a low constant electric field through two or more electrodes inserted in the tissue generating electrolytic products that induce tumor necrosis^11–15^. In Lujan et al.^16^ it is shown that in an EA treatment pH damage is proportional to the applied Coulomb dosage, that is, the electric current multiplied by the time of its application. Also, that an optimal dose-response relationship in terms of pulse number, is the minimum coulomb dosage necessary to achieve total tumor destruction while minimizing healthy tissue damage.

The concept of a dose-response relationship considering pH damage is extended to GET treatments in Lujan et al.^2^ where it is shown that an optimal dose-response relationship in terms of pulse numbers, is the critical pulse dosage yielding maximum reversibly electroporated tissue area with minimal tissue area damage induced by pH fronts. In the present version of the OpenEP, damage due to pH effects is not included. Here, damage refers to IRE effects or temperature excess.

Classical examples of EP-based protocols using the *standard computational* EP * model* (with different electric conductivity definitions) can be found, for instance, in different works^4,5,17,18^, where Laplace’s and Penne’s bioheat equations have been used to describe electric field distribution, temperature, and thermal damage. An extension of the *standard computational* EP * model* model considering tissue capacitance, cell membrane electroporation, and relaxation and resealing between the pulses was introduced in Langus et al.^19^. Results show that the model can predict accurately the time evolution of electric pulses thus being potentially useful for elucidating basic EP mechanisms.

There are two types of software generally used to simulate electroporation protocols: general-purpose simulators such as Matlab, Mathematica, Abacus, OpenFoam, Salome-Meca or COMSOL, among others, and specific purpose simulators such as ApiVizTEP, VISIFIELD, and EView. The former is too general, rather complex, computationally expensive, and definitively requiring substantial knowledge from physics, chemistry, and computational techniques. Nevertheless, COMSOL Multiphysics is an excellent commercial multipurpose simulation tool that is widely used in EP-based protocols. An example of a specific purpose simulator is ApiVizTEP^20^ a pioneering electroporation open-source software toolbox developed at the University of Ljubljana, aiming at the education of researchers and physicians dedicated to these topics. ApiVizTEP can compute and visualize the electric field distribution for different electrode configurations, with real-time interaction. This tool is limited to a two-dimensional domain and uses analytical solutions for obtaining the electric field distribution. Another more advanced specific purpose simulator is VISIFIELD, a web-based software also developed at the University of Ljubljana for the planning of two types of treatments: IRE and ECT. This tool performs automatic tissue segmentation from DICOM medical images to obtain a 3D tissue model. It allows the user to determine the position of the electrodes and the voltage to be applied and generates an easy-to-read and downloadable treatment plan. VISIFIELD has a useful web platform but still is complex and computationally expensive. Recently published, EView (www.eview.upf.edu) is an excellent easy-to-use web-based specific electroporation simulator recently published, developed in Universitat Pompeu Fabra, Barcelona, Spain, in collaboration with Virginia Tech, VA, USA^21^. EView allows the user to set electrode positioning, for any electrode and tissue configuration, on the web browser. The 3D electric field distribution is computed on a dedicated server. According to the authors, EView provides a balance between ease of use and accuracy, aiming at being the initial step among, students, researchers, and clinicians willing to introduce themselves in the field of electroporation. Computations are executed remotely with the logical limitation on the problems that can be tackled. Nevertheless, it provides beginners and experts in the field a quick way to simulate electric field distributions on arbitrary electrode configurations.

Here we introduce OpenEP (https://github.com/LSC-UBA/OpenEP), an specific-purpose simulator for EP-based treatments that is distributed under a free/libre user license. OpenEP provides the EP-based research community with a flexible implementation for predicting the evolution and optimization of several EP-based protocols. It allows also modifying the electrode material and shape: plates or needles dimensions, number of electrodes, anode-cathode distance, pulse polarity, and pulse variability, among others. Moreover, OpenEP describes key physical variables involved in electroporation or pulsed electric field treatments: electric potential, electric field, electrical conductivity, current density, electric current, electric charge, electroporated area or electric field threshold variation in time, and heat distribution. Particularly, the knowledge of the electric field intensity, which is correlated with the electroporated tissue, helps to develop improved strategies to plan and optimize a given treatment. The highly efficient three-dimensional implementation is obtained through the use of C++ and OpenMP on a GNU/Linux environment. This implementation greatly accelerates protocol optimization.

Aside from being open-source, the main difference between the group of general-purpose simulators and OpenEP is that the former, due to its characteristics, it is rather awkward to manipulate when one is searching for optimal EP-based protocols yielding maximum electroporated area with minimum damage. Highly throughput is possible in OpenEP because it possesses a very efficient shared memory implementation that takes advantage of parallel resources, allowing the evolutionary analysis of different complex scenarios by greatly reducing the runtime. The main difference between the group of specific electroporation simulators and OpenEP is that the former is focused on the automatic therapy optimization and generation of a treatment plan and do not consider some of the physical variables related to the EP process and their time evolution and optimization.

OpenEP is validated with theoretical and experimental results from the literature, and its sequential and parallel implementation is thoroughly analyzed. OpenEP is available at https://github.com/LSC-UBA/OpenEP.

This work is organized as follows: section “Methods” presents the *extended standard computational* EP *model*, and the OpenEP software architecture and its implementation; the “Results and discussion” section presents five applications of the OpenEP to typical EP-based protocols and performance analysis. Finally, in the “Conclusions” section, some general conclusions are drawn. Further details of the OpenEP simulator are presented in the supplementary material.

## Methods

As previously discussed, the *extended standard computational* EP * model* consists of replicating for *n* consecutive pulses the *standard computational* EP * model*, via the experimental measurements in time of the successive thresholds. Because experimental data of this threshold variation is generally lacking, an exponential time decay function is assumed based on the few data available. The electric charge conservation equation leading to the *standard computational* EP * model* and its extension, the *extended standard computational* EP * model*, reads:

1 $$\begin{aligned} \nabla \cdot (\sigma \ \varvec{\nabla } \Phi ) = 0 \end{aligned}$$

where $\Phi$ and $\sigma$ are the electrostatic potential and the electrical conductivity, respectively. The electric field *E* is calculated as the gradient of the electrostatic potential, that is,

2 $$\begin{aligned} {\varvec{E}} = - \varvec{\nabla } \Phi . \end{aligned}$$

Electrical conductivity is generally assumed as a function of the electric field and/or temperature. Here, following Arena et al.^5^, the electrical conductivity reads:

3 $$\begin{aligned} \sigma = \sigma _b \,\left( 1 + \alpha \ ( T - T_0 ) \right) \end{aligned}$$

where $\sigma _b$ is the baseline electrical conductivity, $\alpha$ is the coefficient describing the conductivity variations with temperature, and $T_0$ is the initial tissue temperature.

The Penne’s Bioheat equation^18^ is used to compute the tissue temperature:

4 $$\begin{aligned} \rho \ C_p \ \frac{\partial T}{\partial t} = \nabla \cdot (\kappa \ \varvec{\nabla } T ) - {\rho }_b \ {\omega }_b \ C_b \ (T - T_b) + \sigma \ |\nabla \Phi |^2 + q^m \end{aligned}$$

where *T* is the temperature, $\rho$ and $\rho _b$ are the tissue and blood density, respectively; $C_p$ and $C_b$ are the tissue and blood heat capacity, respectively; $\kappa$ is the thermal conductivity; $\omega _b$ is the blood perfusion rate; $T_b$ is the arterial temperature; $q^m$ is the metabolic heat generation; and *t* is the time.

The electric current density *J* (computed through a surface surrounding one of the electrodes), the electric current *I*, and the electric charge *Q* are calculated as follows:

5 $$\begin{aligned} {\varvec{J}} = \sigma {\varvec{E}} \end{aligned}$$

6 $$\begin{aligned} I = \mathop{{\int\!\!\!\!\!\int}\mkern-21mu \bigcirc}{\varvec{J}} \cdot \hat{{\varvec{d}}{\varvec{S}}} \end{aligned}$$

7 $$\begin{aligned} Q= \int _{0}^{t} I dt. \end{aligned}$$

Initial values for the temperature and electrical potential are $T_p$ (expressed in Celsius) and $0 \, \hbox {V}$, respectively. The electrodes (needles or plates) are typically inserted into the tissue or used to hold the tissue. In both cases, a portion of the electrodes is exposed to room temperature and the following convective cooling boundary condition is applied to the exposed portion

8 $$\begin{aligned} - \kappa \ \varvec{{\nabla }} T \cdot \varvec{{\check{n}}} \ = h \ (T - T_{r}) \end{aligned}$$

where *h* is the heat-transfer coefficient, $\varvec{{\check{n}}}$ is a normal vector to the surface of the electrode exposed parts, and $T_r$ is the room temperature.

Each EP-based treatment has an associated set of input variables, such as the number of applied pulses, pulse length (${\hbox {t}}_{ON}$) and frequency, and the voltage-to-distance ratio (V/D) applied between the electrodes. Examples of protocol input parameters are presented in Table 1.

**Table 1:** First column shows the EP-based protocols used; second to fifth column, number of applied pulses, pulse length, frequency, and voltage-to-distance ratio (V/D), respectively; last column shows from which work the data was taken.

EP treatment	No. of pulses	${\hbox {t}}_{ON}$ (s)	Freq. (Hz)	V/D ratio (V/cm)	References
IRE	80	0.0001	1	1800	^5^
ECT	8	0.0001	10	600	^2^
GET	10	0.02	1	300	^22^

During the ON/OFF period the voltage applied at the electrodes is greater than zero or zero, respectively. The ON period usually lasts microseconds or milliseconds, while its ascending voltage ramp takes a few nanoseconds.

Tissue surfaces exposed to the air are modeled through zero flux boundary conditions as shown in Eqs. (9) and (10):

9 $$\begin{aligned} - \kappa \ \varvec{{\nabla }} T \cdot \varvec{{\check{n}}} = 0 \end{aligned}$$

10 $$\begin{aligned} - \sigma \ \varvec{{\nabla }} \Phi \cdot \varvec{{\check{n}}} \ = 0. \end{aligned}$$

### Software architecture and implementation

**Figure 1:**
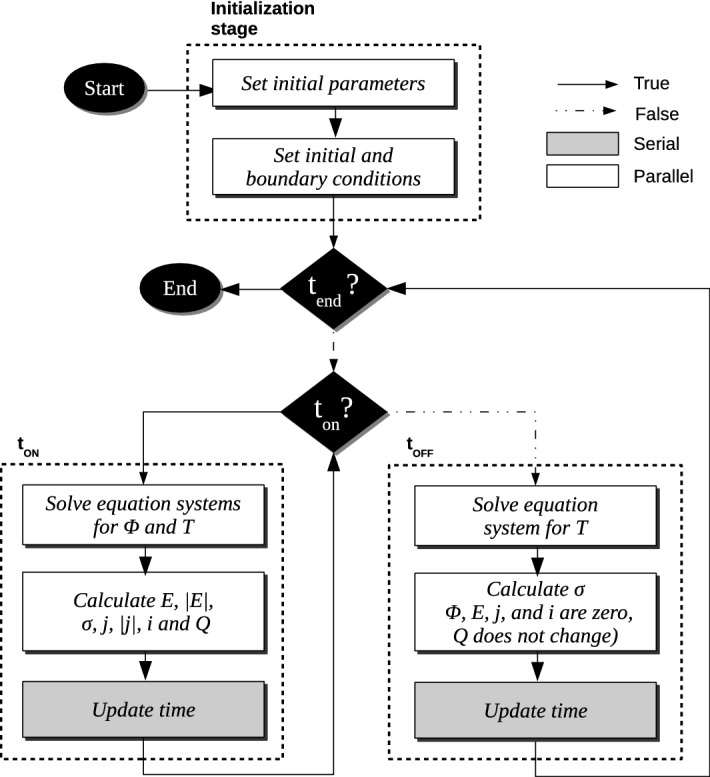
OpenEP flow chart. OpenEP follows a different path when a pulse is being applied to repeat a loop until the end of the simulation is reached.

OpenEP main simulation process is presented in the flow chart of Fig. 1 and implements the *extended standard computational* EP * model* as a sequence of three stages, represented by dashed lines: initialization, $t_{ON}$ and $t_{OFF}$. During the first stage, initialization, all variables are initialized, and the initial and boundary conditions are defined.

The second and third stages, $t_{ON}$ and $t_{OFF}$ respectively, represent a pulse period. The simulation loops over these stages until the maximum number of pulses is reached. Both stages are characterized by internal iterative schemes. In the $t_{ON}$ stage, the Laplace and Penne’s equations associated with the electrostatic potential and the temperature, respectively, are solved. These variables are used for direct calculation of the other physical variables (electric field, electrical conductivity, electrical current density, electric current, and electric charge). During the $t_{OFF}$ stage, the electric field is zero and the Laplace equation is bypassed, with a considerable saving in run time. During this stage, only the temperature and the electrical conductivity are computed. At each stage, output files are saved and time is updated. The chosen output formats were vtk and csv, due to their compatibility with Paraview and several other widely used tools. Code segments associated with most calculations (white blocks) were optimized for multi-processor workstations.

OpenEP is written in C++ and organized in fourteen main source files (see Table 2). General design decision applied to this toolbox, as well as some of the source files (e.g. Makefile or scalar_field.h) were reused from a recently published simulation code: LibreGrowth^23^. Details are presented in the Discussion section.

**Table 2:** Source files description.

Source file	Description
main.cpp	Main simulation process is implemented in this file
electrics_calc.h, electrics_calc.cpp	All electric variables ($\Phi$, ${\varvec{E}}$, $\sigma$, ${\varvec{j}}$, *i*, *Q*) are calculated in these files
temp_calc.h, temp_calc.cpp	Temperature transient solution is solved in these files
par.h	Biological, physical and numerical parameters are declared and/or initialized in this file
mesh.h, mesh.cpp	Mesh class keeps information about geometry and domain discretization. Instances of this class are used in the ScalarField class and VectorField class
scalar_field.h, scalar_field.cpp	ScalarField class is defined in these files. Instances of this class are used in main.cpp, electrics_calc.h, temp_calc.h for depicting $\Phi$, $|{\varvec{E}}|$, $|{\varvec{j}}|$ and $\sigma$
vector_field.h, vector_field.cpp	VectorField class is defined in these files. Instances of this class are used in main.cpp, electrics_calc.h and temp_calc.h for depicting ${\varvec{E}}$ and ${\varvec{j}}$
save.h, save.cpp	Saving of the different scalar and vector fields as well as the log, are implemented in these files

The software was developed for a GNU/Linux system, Ubuntu 18.04.2 LTS (64 bit), running over an Intel Core i5-8250U 8-core CPU at $1.60 \, \hbox {GHz}$ with $7.6 \, \hbox {GB}$ RAM memory. A performance analysis was carried out in this workstation as well as in the computer cluster TUPAC^24^, where each node has four AMD Opteron 6276 (hexadeca core) processors.

Strongly implicit finite difference approximations for solving the *extended standard computational* EP * model* imply the use of many nested loops. These loops were optimized through the shared memory technology OpenMP^25^, through parallel for directives as presented in the following snippet: 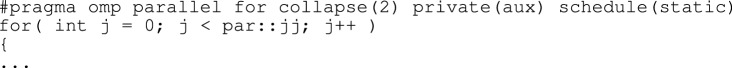


Compilation and execution is made easier using the run.sh bash script. Table S1 in the supplementary material describes the different options controlling the script behavior. Typing in the console: run.sh will create a directory named simulation-000001 with three folders in it: bin, data and src, which stores the simulation executable file, the output data (Paraview-compliant or csv output files) and the source code of this particular simulation, respectively. The next time the script is executed, the simulation directory will be named with the following natural number related to the last simulation, i.e. simulation-000002.

## Results and discussion

OpenEP simulator usage is illustrated with five typical EP treatments from the literature (GET, ECT, HFire and IRE), followed by an OpenEP simulator performance analysis. The configuration file (par.h) for previous protocols is included in the folder cases of the project repository. Domain and numerical parameters are included in the supplementary material, Tables S3 and S4, respectively.

### Gene electrotransfer with two-needle electrodes

The first example is taken from Lacković et al.^4^ presenting simulations of the joule-heating effect in ECT and GET treatments for different electrode geometries. The three-dimensional EP mathematical model was implemented with COMSOL Multiphysics; for geometry modeling and meshing, the MATLAB (Mathworks, Natick, MA) was used.

**Figure 2:**
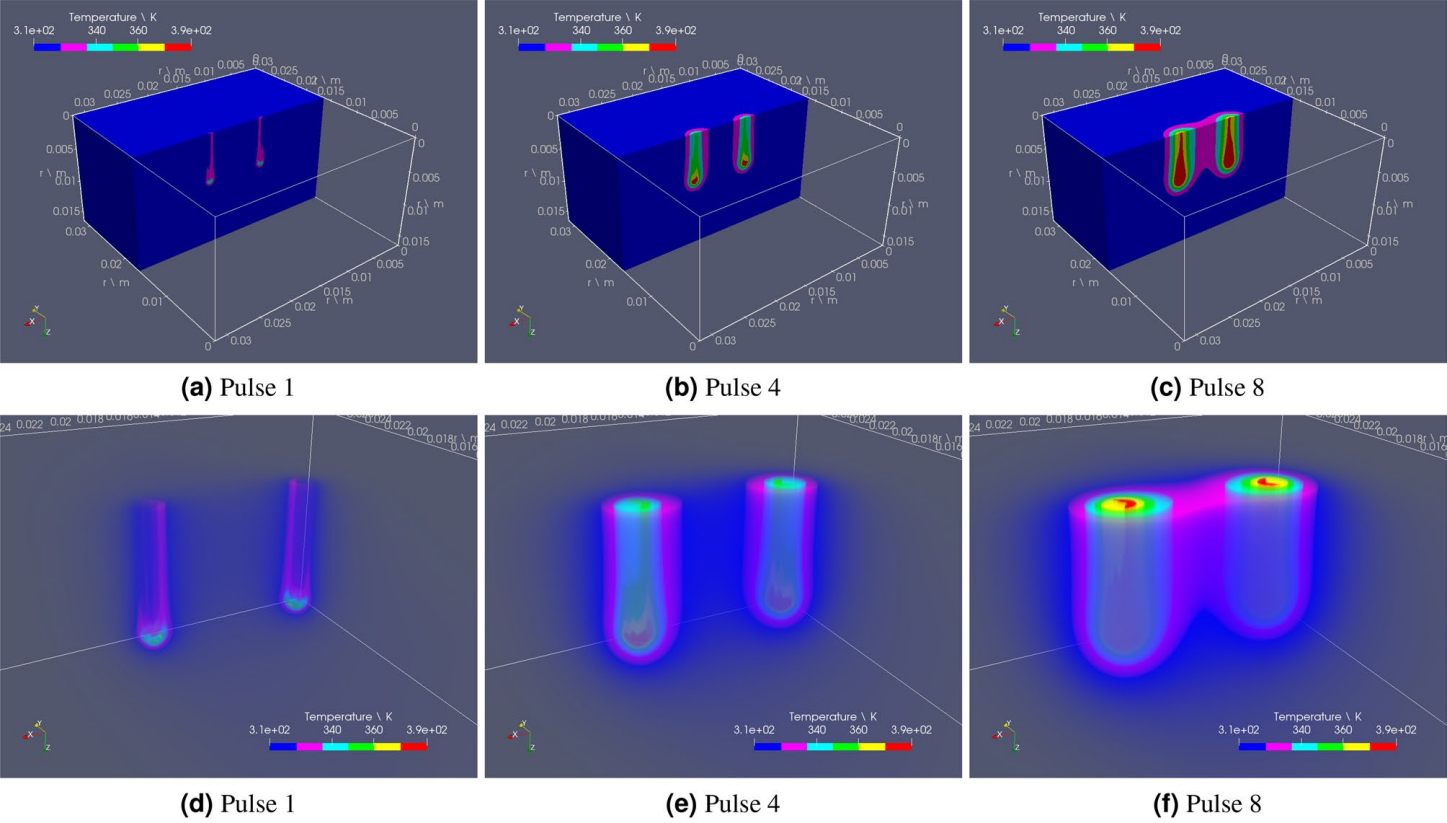
OpenEP predictions of the temperature variation in space for different pulses ($250 \, \hbox {V/cm}$, 8 pulses of $50 \, \hbox {ms}$, $1 \, \hbox {Hz}$). First row shows the temperature distribution; second row is a zoom of the temperature distribution near the electrodes.

The OpenEP simulator mimics the GET protocol with needle electrodes presented in Fig. 9 from Lacković et al.^4^. The set of parameters for model description is defined in the file par.h. The panel in Fig. 2 shows simulations of the temperature distribution at different times. Figure 2a–f depict temperature during the ON stage of pulses 1, 4 and 8, respectively. The box represents a tissue portion and the cylinders, the electrodes. Whereas bluish areas are close to tissue temperature ($37 \, ^{\circ }\hbox {C}$), near the electrodes (particularly in the tips) temperature is higher, mainly because of the influence of the joule-heating effect.

**Figure 3:**
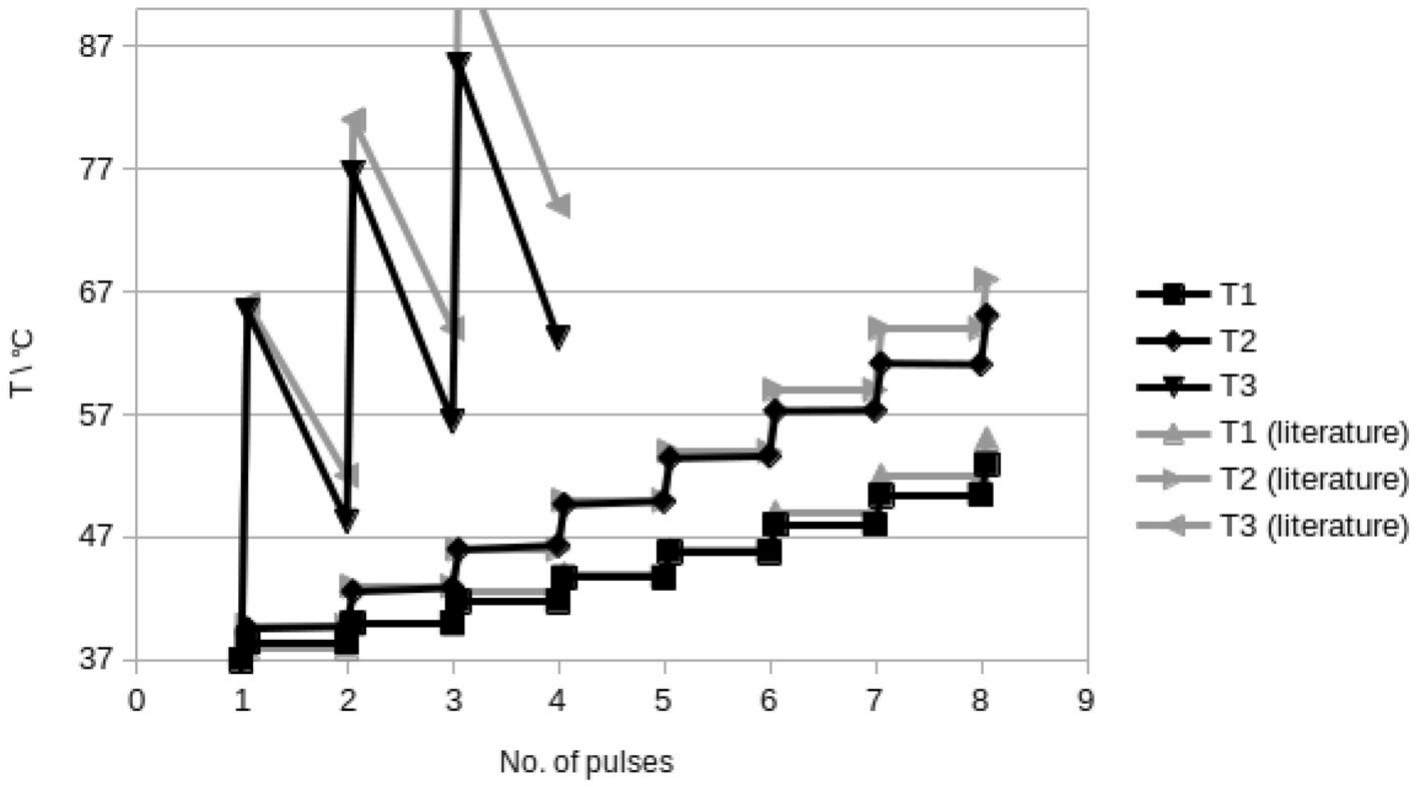
OpenEP prediction of the temperature versus time for a protocol of $250 \, \hbox {V/cm}$, 8 pulses of $50 \, \hbox {ms}$, $1 \, \hbox {Hz}$. OpenEP results (black lines), literature results^4^ (grey lines). Temperature is computed and recorded at different locations: close to the electrode (T1), in the quarter (T2), and the middle of the inter-electrode distance (T3).

Figure 3 presents a comparison between OpenEP results and those in Fig. 9 from Lacković et al.^4^. As shown in Table S3 in the supplementary material, the simulation was performed for needle electrodes during a train of eight pulses ($250 \, \hbox {V/cm}$, $50 \, \hbox {ms}$, $1 \, \hbox {Hz}$) with an electrical conductivity of $0.504 \, \hbox {S/m}$. The temperature distribution was calculated and recorded at different locations: close to the electrode (T1), in the quarter (T2), and the middle (T3) of the distance between electrodes. OpenEP temperature results at locations above-mentioned are close with those in Lackovic et al.^4^.

### Electrochemotherapy and irreversible electroporation

**Figure 4:**
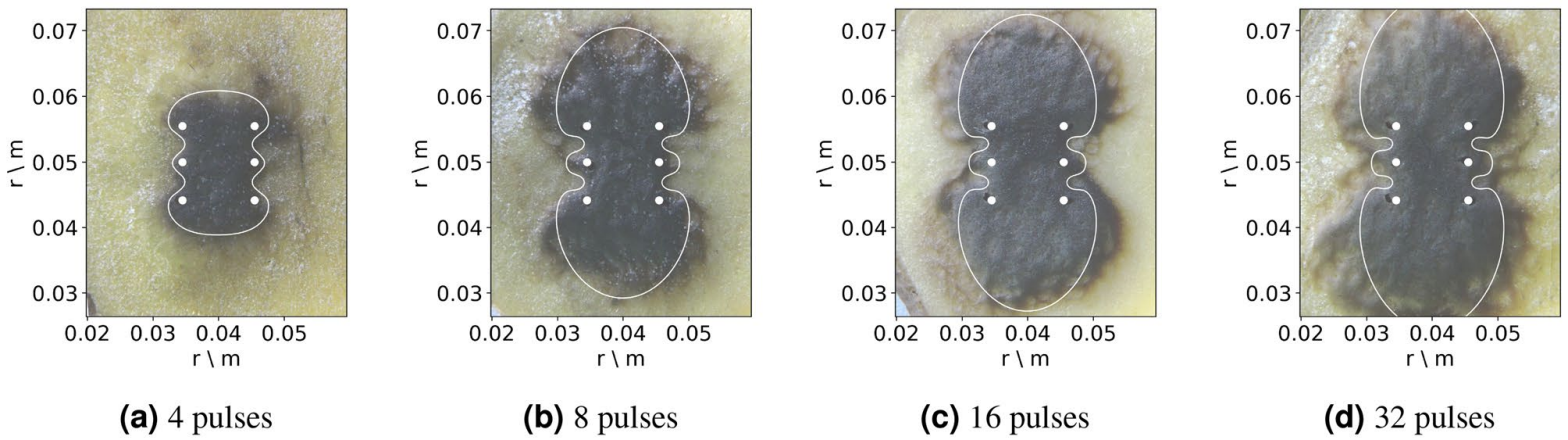
Experimentally measured electroporated area (dark area) versus pulse number for an EP-based protocol (4, 8, 16 and 32 pulses of $100 \, \upmu \hbox {s}$, $1 \, \hbox {Hz}$ with an amplitude of $1500 \, \hbox {V}$), and superposed, the predicted threshold variation versus pulse number obtained with the OpenEP simulator (Figure taken from Marino et al.^10^).

Here, the goal of the OpenEP is to simulate the time evolution of an EP-based protocol (ECT and IRE) from Suarez et al.^3^. The in vitro model consists of the application of different EP-based protocols with an arrangement of six electrodes inserted in a vegetable tissue (potato slice). After the treatment, the tissue near the electrodes is gradually darkened due to an enzyme oxidation process. This darkened area is generally considered a sign of electroporated tissue. A series of measurements in time of the electric current, temperature, electrical conductivity, and the darkened area, for different electric fields ($500 \, \hbox {V/cm}$, $1000 \, \hbox {V/cm}$, $1500 \, \hbox {V/cm}$) are presented. The in-silico model uses the *standard computational* EP * model* with the tissue electrical conductivity made a function of the electric field, the temperature, and the pulse number. Computational results using COMSOL, and Fortran codes developed by the authors were presented. Experimental measurements and simulations show that the electroporated dark potato area increases logarithmically with pulse number, the electric field remains constant, and the current density and the conductivity increase at the same rate (Figs. 2, 3, 4 of the above-mentioned work).

To simulate the time evolution of previous results, Fig. 4 taken from Marino et al.^10^, presents a graph of the experimentally measured electroporated area (dark area) versus pulse number for an EP-based protocol (4, 8, 16, and 32 square pulses of $100 \, \upmu \hbox {s}$, $1 \, \hbox {Hz}$ with an amplitude of $1500 \, \hbox {V}$), and superposed, the predicted threshold variation versus pulse number obtained with the OpenEP simulator, using the *extended standard computational* EP * model*. Comparison between predicted and experimental results serves to validate the ability of the OpenEP simulator to predict the EP-based treatment time evolution.

**Figure 5:**
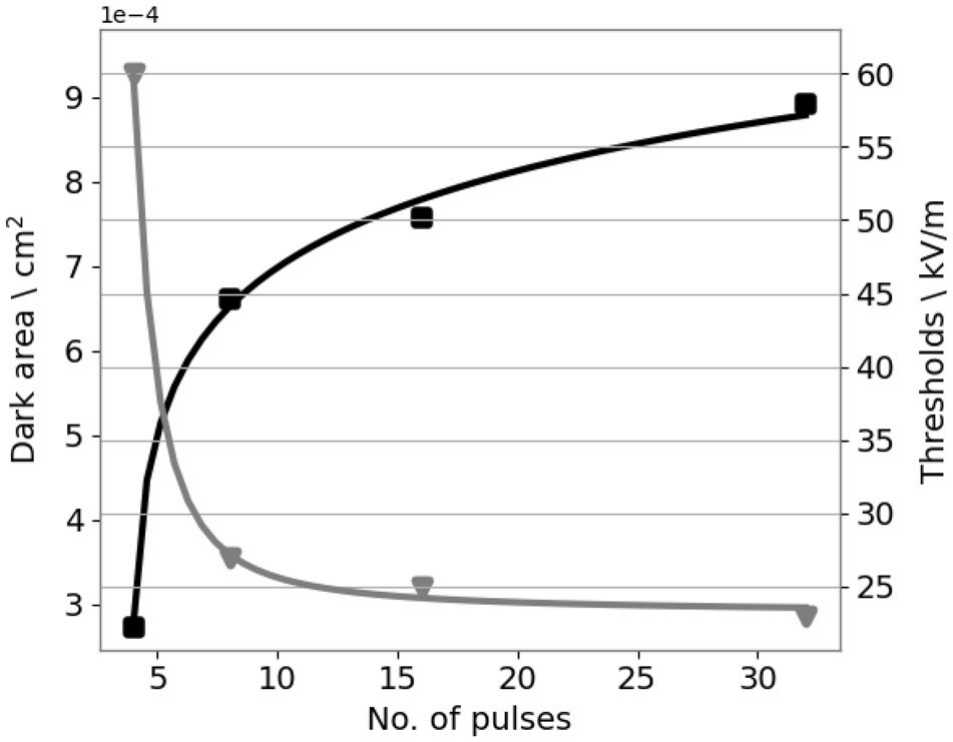
Electroporated area versus pulse number (black line); threshold versus pulse number (grey line). Figure taken from Marino et al.^10^.

As discussed in the introduction, the *extended standard computational* EP * model* is based on the assumption of an exponential time decay function for the threshold variation^2^. To justify this assumption, firstly, the variation of the experimentally measured electroporated area (darkened area) from the previous figure data can be plotted, and then, using the *extended standard computational* EP * model*, the predicted threshold can be plotted. The result is presented in Fig. 5 demonstrating that the threshold has an exponentially time decay form, thus constituting a sound theoretical assumption.

**Figure 6:**
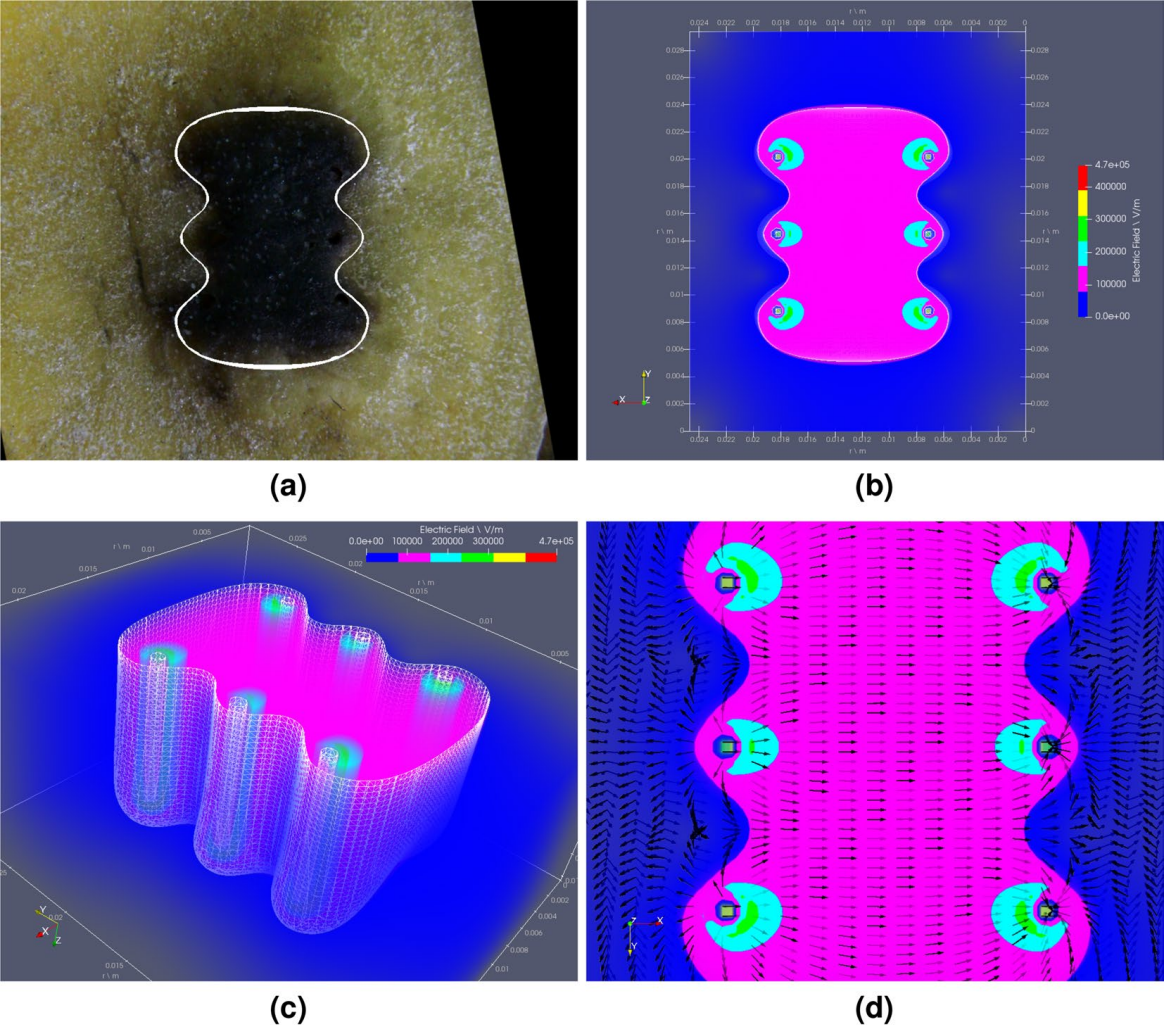
OpenEP predictions of: (**a**) Electroporated potato area (darkened area with a white contouring line showing the threshold) after four pulses (${100} \, {\upmu }\hbox {s}$, $1 \, \hbox {Hz}$ and $1500 \, \hbox {V/cm}$); (**b**) Top slice of the electric field distribution showing the electric field threshold after the fourth pulse (pink area); (**c**) Electric field distribution seen as a 3D volume rotated to right; and (**d**) Vector field representation of the field distribution.

For completeness and as illustration of the results and type of graphic display available in the OpenEP, next figure presents simulations of the experimental and computational results presented in Suarez et al.^3^. Figure 6 shows (a) Electroporated potato area (showing the threshold) after four pulses ($100 \, \upmu \hbox {s}$, $1 \, \hbox {Hz}$ and $1500 \, \hbox {V/cm}$); (b) Top slice of the electric field distribution showing the electric field threshold after the fourth pulse (pink area); (c) Electric field distribution is seen as a 3D volume rotated to the right; and (d) Vector field representation of the electric field distribution.

### Electrochemotherapy and electrogenetransfer

Here, OpenEP is used in the search of an optimal EP-based treatment (example taken from the experimental results from Sel et al.^17,26^). It is recalled that an optimal EP-based protocol, such as ECT or GET, is a function of pulse amplitude, length, number, and frequency, among other variables. Moreover, in Lujan et al.^2^ it is shown that an optimal dose–response relationship in a GET protocol is the critical pulse dosage yielding maximum reversibly electroporated tissue area with minimal tissue area damage induced by pH fronts. In the present version of the OpenEP, damage refers to IRE effects or temperature excess.

Figure 5 from Sel et al.^26^ shows experimental irreversible electroporated thresholds fronts (white lines contouring an area of tissue necrosis) and the associated simulated thresholds (dark areas) for three ECT protocols ($\hbox {U}=960 \, \hbox {V}$, n $=$ 8, $1 \, \hbox {Hz}$, and $100 \, \upmu \hbox {s}$). Figure 5a reveals that the threshold fronts surrounding the electrodes barely cover the target, defined as the area between electrodes (but not shown). While in Fig. 5b, threshold fronts coalesce covering a larger area of the target, in Fig. 5c the target is almost fully covered. This shows that with a constant pulse voltage, increasing electrode diameters, electroporation increases. It also reveals that by tuning electrode diameter, optimal electroporated area coverage can be attained. A similar effect (increasing the electroporated area) is reached, by increasing pulse number (as shown in the potato example above). Focusing on Fig. 5c, for example, it is possible to attain optimal electroporated area coverage by tuning the pulse number. This can be done (as described in the OpenEP simulations of the potato model), computing the electroporated area with the assumption of an exponential time decay threshold. Within the window of pulse numbers defining reversible and irreversible thresholds, there is an optimal pulse dosage in which the electroporated area is a maximum with minimum damage. Here, damage refers to IRE effects.

#### High-frequency irreversible electroporation (H-FIRE) alternating polarity pulses

The third example is taken from Sano et al.^27^ presenting in-silico and in-vitro studies of H-FIRE bursts of ultrashort ($0.25\, \upmu \hbox {s}$ and $5\, \upmu \hbox {s}$) alternating polarity pulses that produce more predictable ablations and alleviate the associated IRE muscle contractions. A shortcoming of H-FIRE is the ablation of smaller volumes of tissue than IRE. The results in Sano et al.^27^ show that asymmetric H-FIRE waveforms can be used to create ablation volumes equivalent to standard IRE treatments. The in-vitro model consists of the application of different IRE (H-FIRE) protocols in three experiments in cell cultures to evaluate the implications of pulse asymmetry in electroporation therapies. In the in-silico model, the reversible and lethal thresholds found in vitro are incorporated into a three-dimensional finite element model solved with the COMSOL software. The aim is to predict the size and shape of ablations which would be created by H-FIRE waveforms if $3 \, \hbox {kV}$ pulses were delivered into live tissue through clinical electrodes (these models were then validated against ablations created in ex vivo liver tissue).

**Figure 7:**
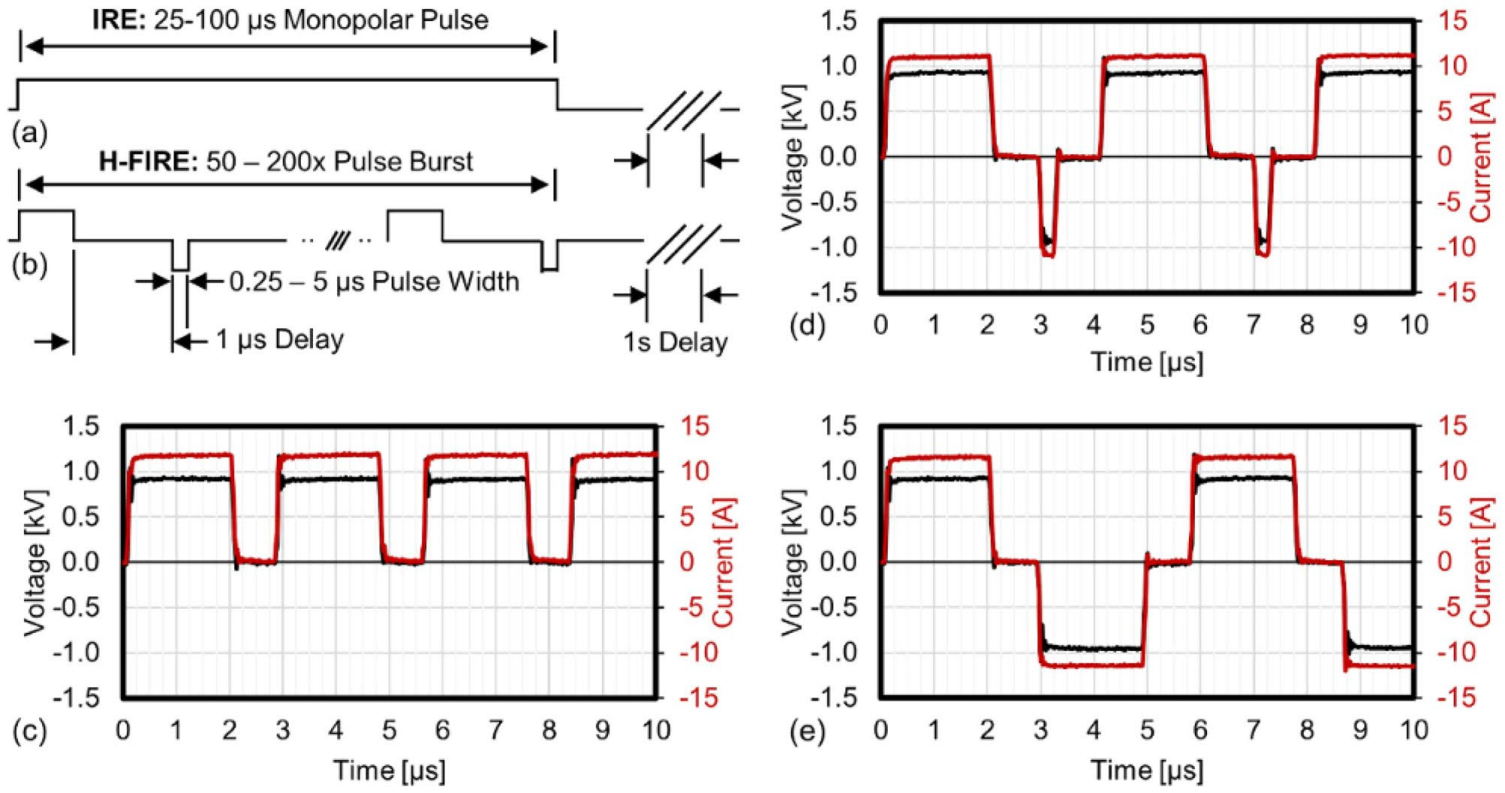
Examples of IRE and Different H-FIRE Pulse Waveforms: (**a**) IRE treatments deliver a series of long-duration monopolar pulses. (**b**) H-FIRE treatments typically deliver multiple bursts containing $0.25\, \upmu \hbox {s}$ and $5\, \upmu \hbox {s}$ alternating polarity pulses. Example waveforms showing the first $10 \, \upmu \hbox {s}$ of a (**c**) $2 \, \upmu \hbox {s}$ monopolar, (**d**) asymmetric $2\, \upmu \hbox {s}$, $1\, \upmu \hbox {s}$ and $0.25\, \upmu \hbox {s}$ (positive–delay–negative), and (**e**) symmetric $2\, \upmu \hbox {s}$, $1\, \upmu \hbox {s}$ and $2\, \upmu \hbox {s}$ (positive–delay–negative) H-FIRE bursts. Figure reproduced from Sano et al.^27^.

Here, the OpenEP simulator is used to predict the results in Sano et al.^27^ of an H-FIRE protocol in a well with two electrodes of $1 \, \hbox {mm}$ diameter separated $3 \, \hbox {mm}$ and bipolar asymmetric pulse bursts. Figure 7 taken from Sano et al.^27^ explains the type of pulses being applied (Fig. 7).

**Figure 8:**
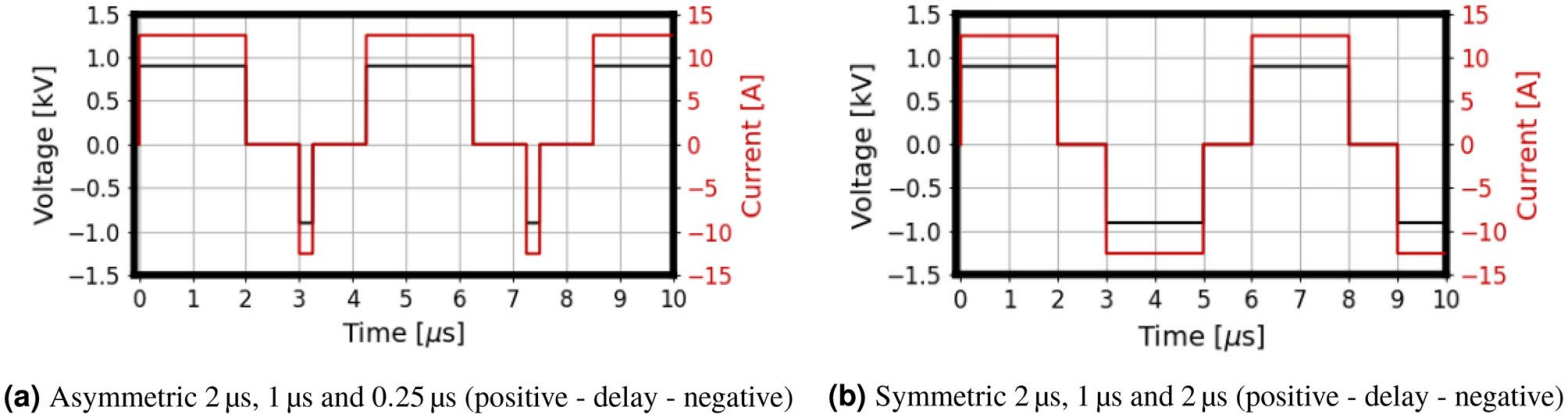
OpenEP prediction of the first $10 \, \upmu \hbox {s}$ of different H-FIRE Pulse Waveforms shown in the previous figure.

Figure 8 shows the OpenEP simulator reproduction of the first $10 \, \upmu \hbox {s}$ of different H-FIRE Pulse Waveforms shown in the previous figure: (a) asymmetric $2\, \upmu \hbox {s}$, $1\, \upmu \hbox {s}$ and $0.25\, \upmu \hbox {s}$ (positive–delay–negative) and (b) symmetric $2\, \upmu \hbox {s}$, $1\, \upmu \hbox {s}$ and $2\, \upmu \hbox {s}$ (positive–delay–negative) H-FIRE bursts. A comparison with the results in the previous figure serves to validate the OpenEP ability to simulate symmetric and asymmetric bipolar pulses.

**Figure 9:**
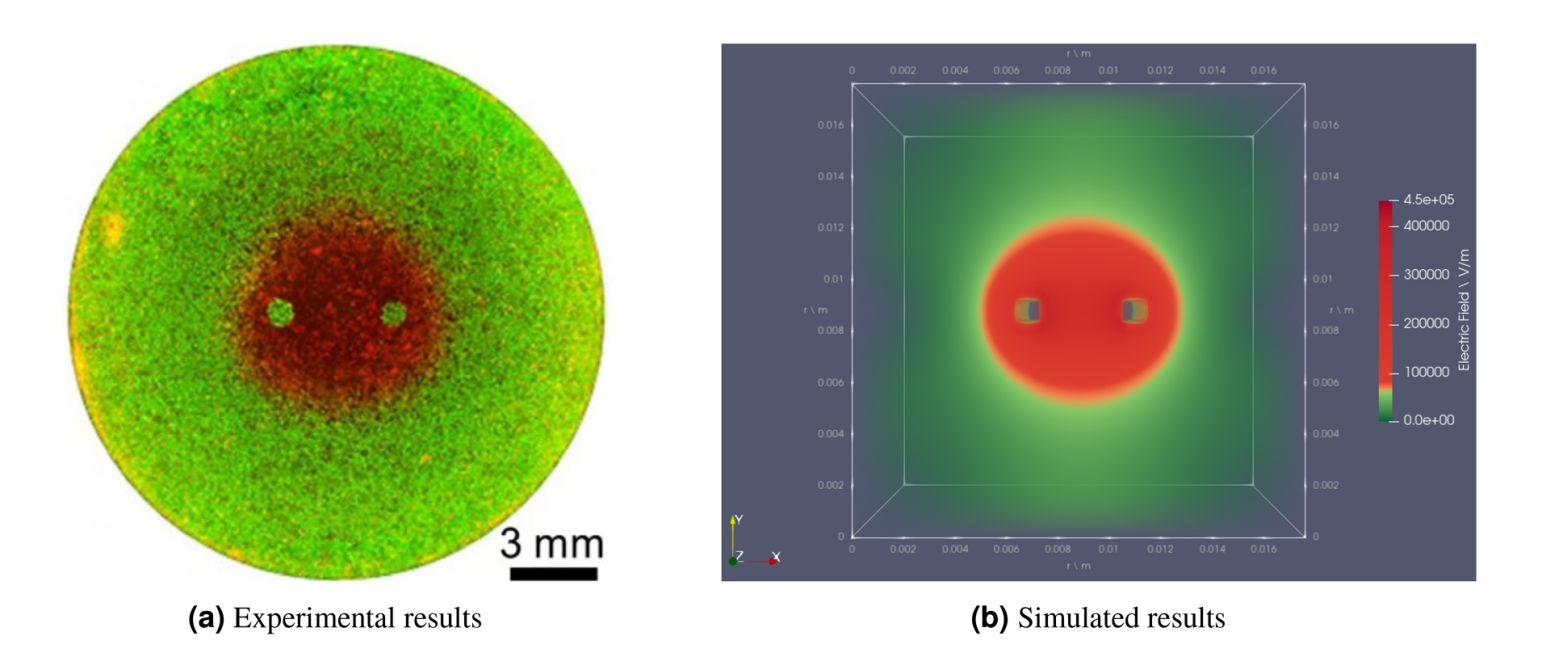
(left) Experimental measurement of electroporated [Red] and unaffected [Green] MDA-MB-231 BR3 cells immediately post-treatment after exposure to 100x H-FIRE Bursts ($2\, \upmu \hbox {s}$, $1\, \upmu \hbox {s}$ and $0.5\, \upmu \hbox {s}$) energized for $50 \, \upmu \hbox {s}$ (figure taken from Sano et al.^27^); (right) OpenEP prediction of the electric field distribution (the well and the $1 \, \hbox {mm}$ electrodes, spaced $3 \, \hbox {mm}$ edge-to-edge, used to deliver $900 \, \hbox {V}$, are approximated by a cube with a uniform cubic mesh). The $500 \, \hbox {V/cm}$ isoline separating red and green zones indicate the irreversible electric field threshold.

Figure 9a shows the experimental results taken from Sano et al.^27^ consisting of electroporated (red) and unaffected (Green) MDA-MB-231 BR3 (human breast cancer) cells after the exposure to 100 × H-FIRE bursts. Each burst used a $2\, \upmu \hbox {s}$, $1\, \upmu \hbox {s}$, $0.5\, \upmu \hbox {s}$ waveform (positive-delay-negative time) which was energized for $50 \, \upmu \hbox {s}$. Figure 9b shows the OpenEP simulator electric field distribution prediction. Again, a comparison with the results from Sano et al.^27^ validates the OpenEP ability to simulate asymmetric H-FIRE bursts electric field distributions.

#### Electrochemotherapy and irreversible electroporation with plate electrodes

The fourth example of an EP-based protocol using two-plate electrodes is again taken from Lackovic et al.^4^. The aim of this example is merely to show more visualizing capabilities of the OpenEP for electroporation variables not shown in previous examples.

**Figure 10:**
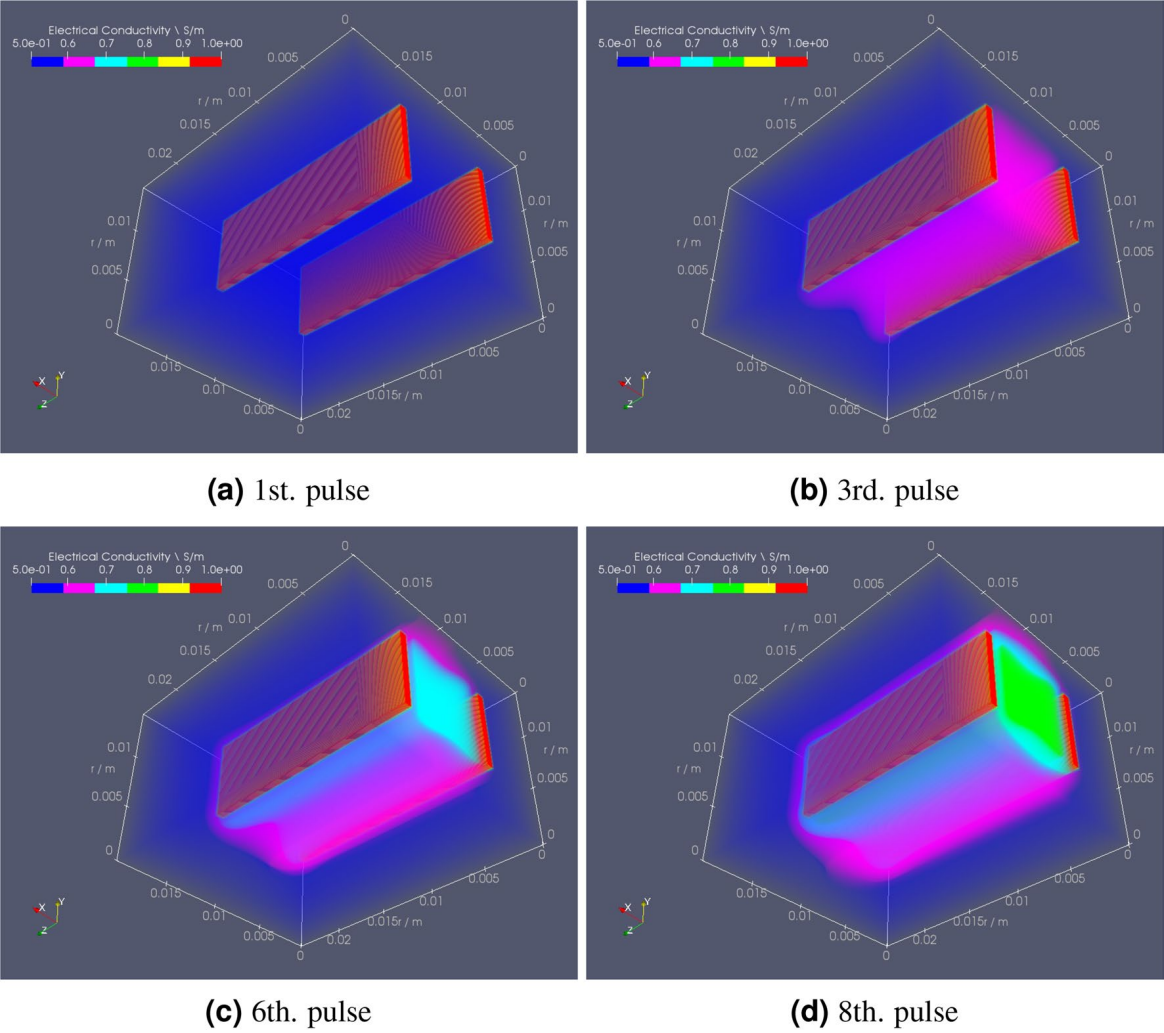
OpenEP predictions of the electrical conductivity variation ($\sigma$) at different pulses ($250 \, \hbox {V/cm}$, 8 pulses of $50 \, \hbox {ms}$, $1 \, \hbox {Hz}$).

**Figure 11:**
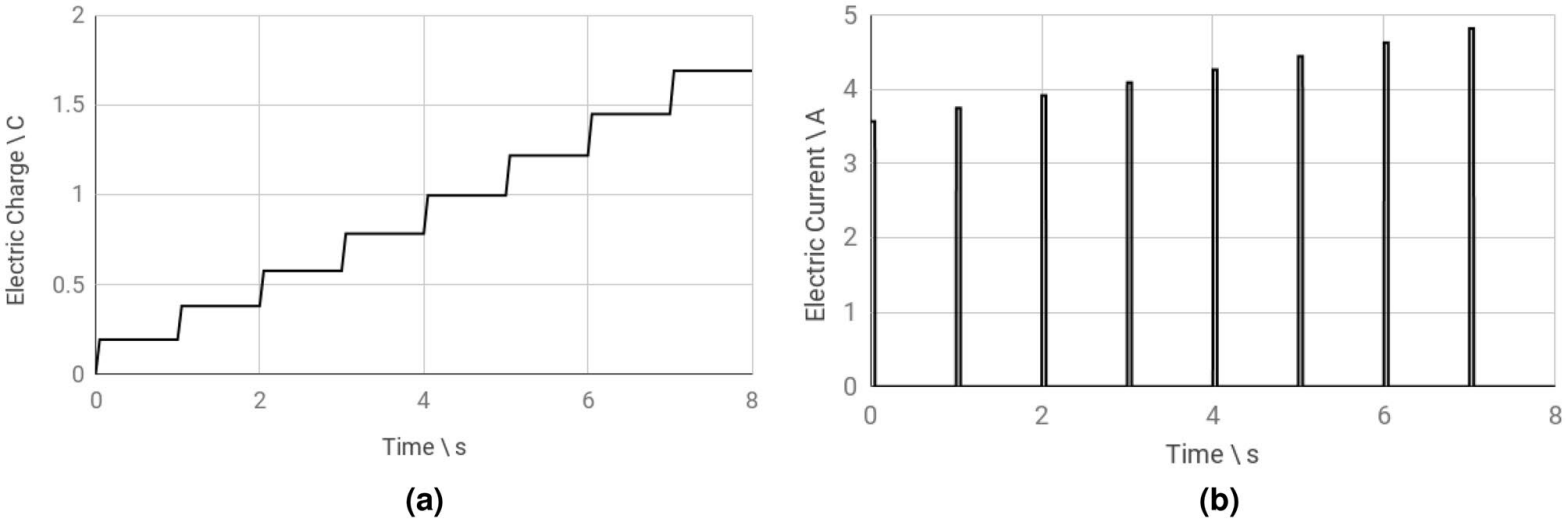
OpenEP predictions of: (**a**) electric charge versus time; (**b**) electric current versus time ($250 \, \hbox {V/cm}$, 8 pulses of $50 \, \hbox {ms}$, $1 \, \hbox {Hz}$).

**Figure 12:**
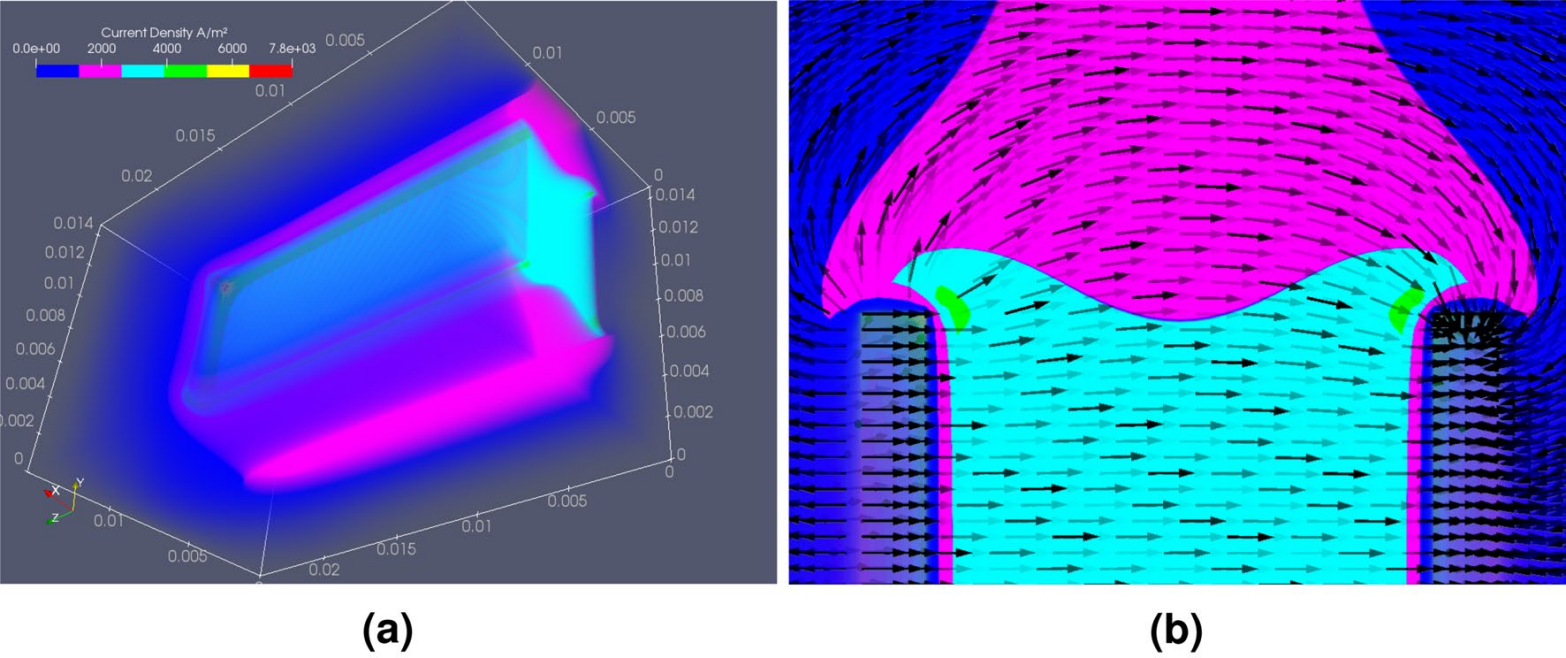
OpenEP predictions of: (**a**) Snapshot of the electric current density distribution ($250 \, \hbox {V/cm}$, 8 pulses of $50 \, \hbox {ms}$, $1 \, \hbox {Hz}$); (**b**) snapshot of the top slice of the electric current density vector field representation.

The following OpenEP results simulates a protocol of eight pulses ($250 \, \hbox {V/cm}$, 8 pulses of $50 \, \hbox {ms}$, $1 \, \hbox {Hz}$), with a two plate electrode of $19 \times 7 \times 1 \, \hbox {mm}$, separated by $5 \, \hbox {mm}$. Figure 10 shows snapshots of the electrical conductivity ($\sigma$) distribution at four different times. Results show that $\sigma$ increases with the number of pulses. Moreover, near the electrodes, $\sigma$ is much larger than away from them (see Table S.3 in the supplementary material). Thus, the color bar is saturated to $1 \, \hbox {S/m}$, allowing the capture of minor differences found away from the electrodes. As mentioned above, electroporation causes an increment in conductivity which in turn causes an increase in the electric current. This can be seen in Fig. 11b, where this variation is shown over time. Figure 11a presents electric charge (*Q*) vs time. As no current flows during the OFF period, no changes occur in *Q* during that cycle. Seen as a 3D volume rotated to the right, current density norm distribution is depicted in Fig. 12a. Finally, Fig. 12b illustrates a top slice of its vector field representation.

### Performance analysis

**Figure 13:**
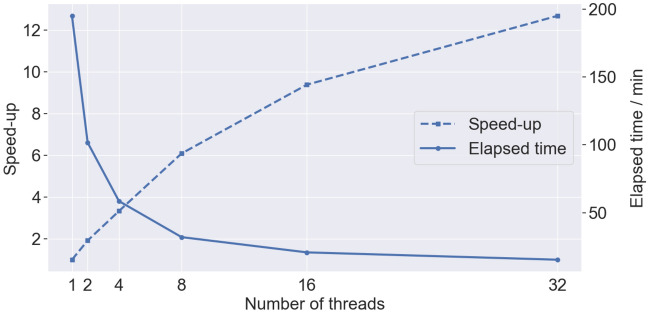
Speed-up (dashed line) and elapsed time (solid line) versus thread usage.

The TUPAC computer cluster was used to perform a strong scalability analysis (a multiprocessor expansion with a fixed-size problem) of the OpenEP simulator applied to a GET protocol. The TUPAC computer cluster is equipped with 64 nodes of $2.3 \, \hbox {GHz}$. Compiler g++ version 6 was used to generate the executable file. To analyze OpenEP simulator scalability when the number of threads was increased, the OpenMP speed-up was used. The following results were obtained when the OpenEP simulator was applied to a GET protocol (8 pulses of 50 ms, 250 V/cm, 1 Hz, a 3D uniform grid of 907,000 nodes, 80,000 time steps of 0.05/1000 during ON-stage, and 0.95 during OFF stage). In Fig. 13, the dashed line shows that with 32 threads the single-core simulation can be outperformed up to 13*X*. The solid line shows the running time decreasing from $195$ to $15 \, \hbox {min}$. The node architecture has a strong memory bottleneck in the memory access, which means that the efficient use of the available parallel resources is extremely difficult. Most of the applications running in this cluster only use 24 cores per node; increasing this number also increases the total running time. In all cases, simulations were carried out in triplicate, choosing those with lower execution times to decrease the interference with other processes running in the assigned computer node of the cluster.

**Figure 14:**
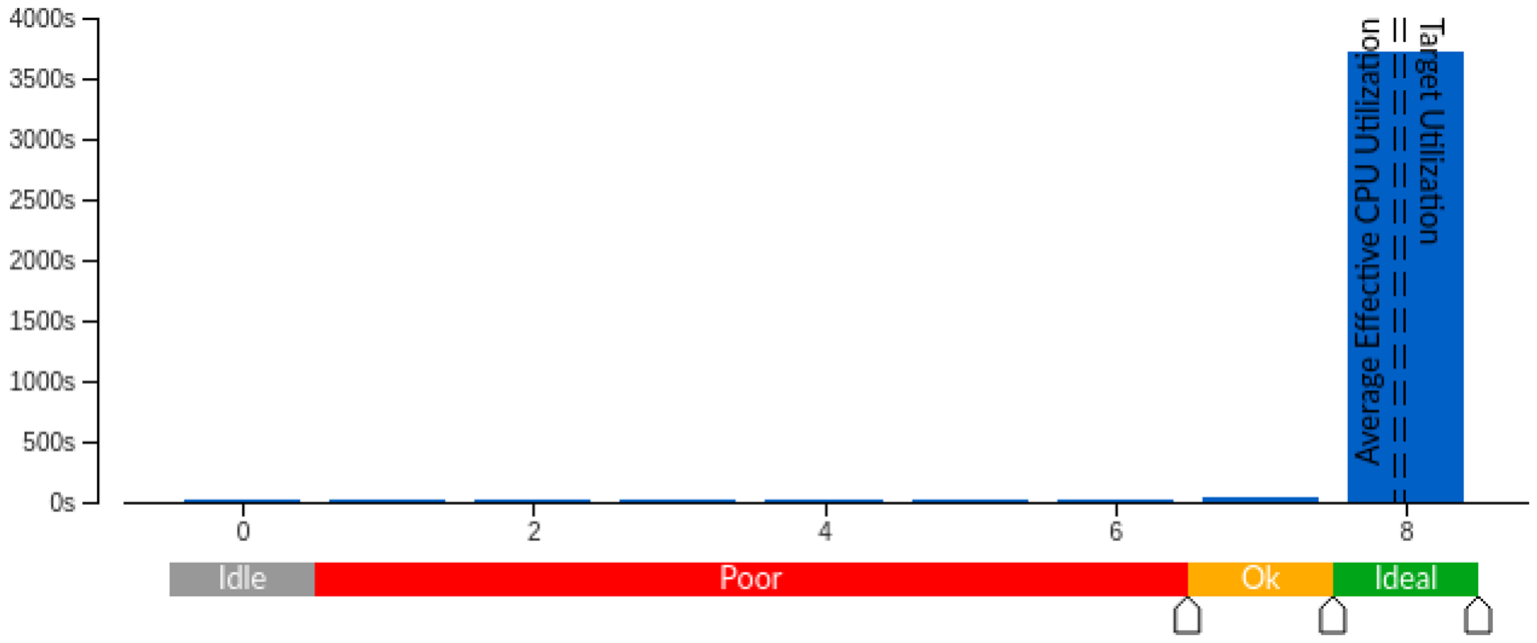
Effective CPU utilization histogram.

Furthermore, OpenEP profiling under GNU/Linux was done on a different workstation. Intel VTune Amplifier^28^ was used to analyze the program execution. Eight threads were used for matching the maximum number of logical cores. The elapsed and CPU times were $63.11 \, \hbox {min}$ $500.44 \, \hbox {min}$, respectively. In Fig. 14, the effective CPU utilization is presented, showing that the majority of the time OpenEP runs in eight logical cores simultaneously.

**Figure 15:**
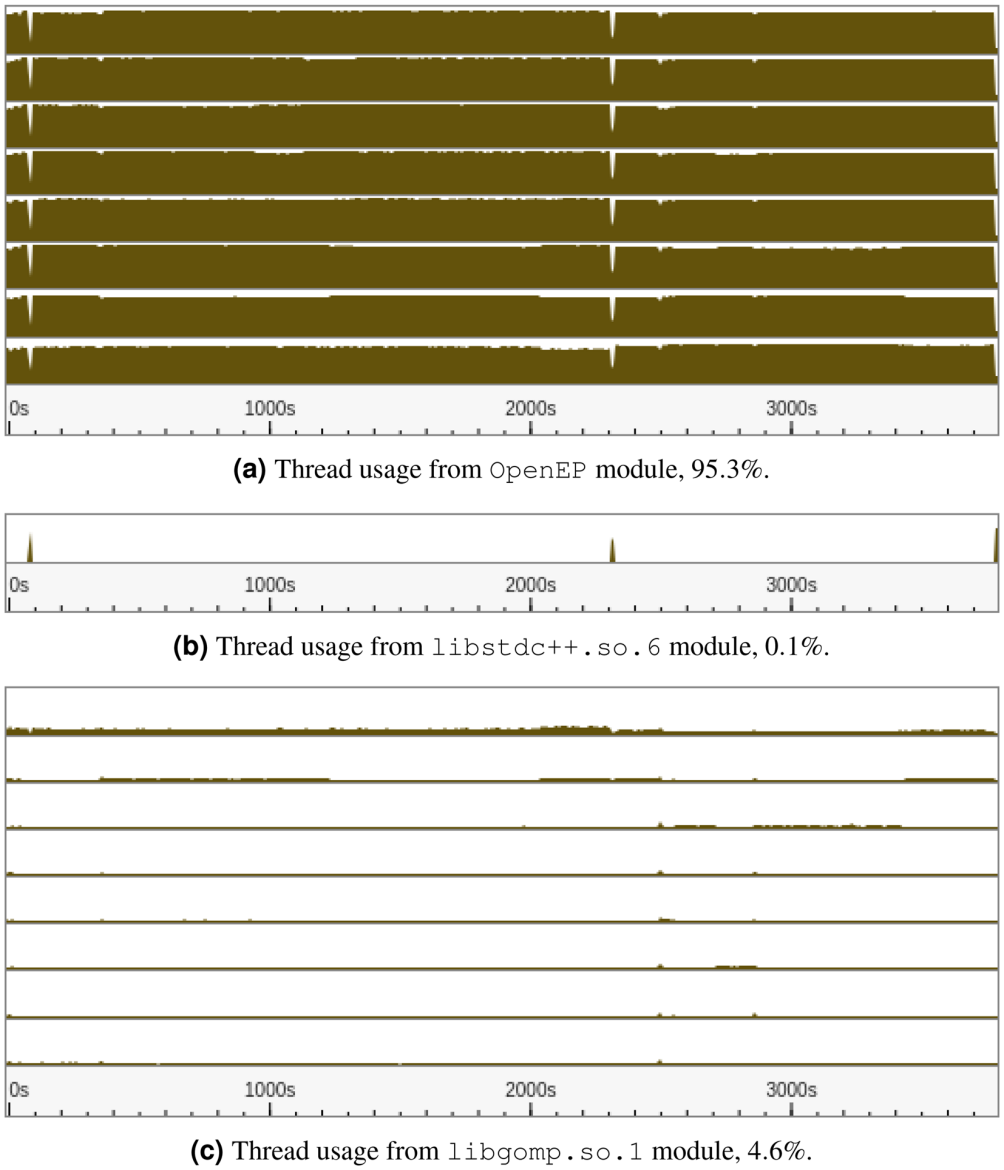
CPU thread usage versus time, for the different program modules. Brown areas represent CPU usage and white areas the running state.

Finally, in Fig. 15, CPU thread usage is shown for all the program modules. Brown areas represent CPU usage and white areas the running state. This information is disaggregated by the main program modules: Fig. 15a–c show modules OpenEP, libstdc++.so.6 and libgomp.so.1 respectively. As observed, OpenEP makes intensive use of the hardware resources, using more than $95\%$ of the CPU.

OpenEP implements typical EP-based treatments used in a wide variety of scientific publications^3–5,8,29,30^. Thanks to its physical dynamics modeling capacity, OpenEP constitutes a valuable tool for seeking the optimal combination of electrode geometries, field intensity, pulse length, heat distribution, and conductivity to improve the efficiency of the treatments. In particular, maximizing electroporated volume and minimizing damaged tissue is a key point in tumor therapies like ECT or GET.

Presently, to the best of our knowledge, there are no FLOSS (Free/Libre Open Source Software) simulation codes available specialized in EP modeling. Unlike the alternatives presented in the Introduction section (except for the educational 2D software ApiVizTEP), the OpenEP is Open Source software and it is licensed under the GNU Public License, V3. Its code is freely available at^31^ and modification, as well as redistribution, are allowed. The targeted audience of OpenEP is the EP-based research community. The package in its present state allows researchers a flexible implementation for predicting the evolution and optimization of several EP-based protocols, a facility not available in other packages. Of course, some programming knowledge is required on the part of the researcher. As in other packages, OpenEP allows manual modification of the electrode material and shape: plates or needles electrodes, number of electrodes, anode-cathode distance, number of pulses along with the polarity, frequency, width, and amplitude, among others. However, it lacks a graphical interface. Certainly, the present version is not attractive for beginners or clinicians. However, there is the intention to improve it by adding a graphical interface, a mesh generation module, a pH damage module, and MPI capabilities for parallel processing, among other improvements.

As previously mentioned in the “Methods” section, software architecture decisions were based on the simulation code LibreGrowth^23^. This software was used for describing the micro-environmental influence on micro-tumor infiltration patterns through in-silico/in-vitro experimentation^32–34^. A possible future work could be the combined use of both modules for modeling the tumor proliferation and invasion into the peripheral host tissue and its treatment with EP-based tumor treatments.

Finally, as the scientific community continues to increase its interest in EP-based treatments, in-silico modeling, as well as its interactions with in-vivo/in-vitro experimentation, will continue to grow in importance. By making this application freely available, it is expected to contribute to the field of EP research and its applications.

## Conclusions

We present OpenEP, a specific purpose simulator for EP-based tumor treatments that is distributed under a free/libre user licence. It brings to researchers in the field a flexible implementation for predicting the evolution and optimization of several EP-based treatments. OpenEP is based on the numerical solution of the nonlinear Laplace’s equation for the electric field, Pennes’ Bioheat equation for the temperature, and the *extended standard computational* EP * model*; it uses finite differences on a three-dimensional uniform grid and standard relaxation procedures. OpenEP allows the customization of tissue type; electrode geometry and material; pulse type, intensity, length, and frequency.

OpenEP is validated with several theoretical and experimental results from the literature and applied to five typical EP-based treatments: GET with two-needle electrodes, ECT and IRE with four-needle electrodes, ECT and GET with two-needle electrodes; H-FIRE bipolar asymmetric pulse bursts with two-needle electrodes, and IRE with plate electrodes. Results show that the OpenEP facilitates the prediction of an optimal EP-based tumor treatment, such as ECT or GET, defined as the critical pulse dosage yielding maximum electroporated tissue with minimal damage.

The OpenEP code is implemented in C++ for GNU/Linux systems and optimized through the OpenMP shared memory technology. Its sequential and parallel implementation performance is thoroughly analyzed, showing speedups of more than one order of magnitude.

OpenEP displays a highly efficient shared memory implementation by taking advantage of parallel resources; this permits a rapid prediction of EP-based tumor treatment efficiency by pulse number tuning. The OpenEP is a readable and easy-to-modify implementation allowing code adaptation to a broad variety of treatment configurations; its output is readily analyzed with the powerful visualization toolkit Paraview.

Despite the availability of several general and specific tools modeling the electroporation phenomenon, currently, there are no openly-available tools specifically designed to predict and optimize this complex phenomenon, which can also be used as a platform for therapy optimization.

## Supplementary information

Supplementary Informations.

## Data Availability

The simulation code is available in https://github.com/LSC-UBA/OpenEP under a GPL v3 software licence.
