# Supplementary material for: OpenEP: an open-source simulator for electroporation-based tumor treatments
Source: Sci Rep. 2021 Jan 14;11:1423. doi: 10.1038/s41598-020-79858-y (PMC7809294; doi:10.1038/s41598-020-79858-y)
Supplement: Supplementary file 1 — Supplementary Informations. [file 41598_2020_79858_MOESM1_ESM.pdf]

Supplementary Material

OpenEP: an open-source Simulator for Electroporation-based Tumor Treatments

M. Marino, E. Luján, E. Mocskos, and G. Marshall

December 1, 2020

Numerical model

In what follows some details of the numerical model discussed in the Methods section are presented.

The non-linear Laplace's equation (Eq. 1) has three terms:

$$\frac{\partial}{\partial x}\left(\sigma \frac{\partial \Phi}{\partial x}\right) + \frac{\partial}{\partial y}\left(\sigma \frac{\partial \Phi}{\partial y}\right) + \frac{\partial}{\partial z}\left(\sigma \frac{\partial \Phi}{\partial z}\right) = 0 \tag{A}$$

The first term is discretized with the following central difference scheme:

$$\left(\left(\sigma \frac{\partial \Phi}{\partial x}\right)_{i+\frac{1}{2},j,k} - \left(\sigma \frac{\partial \Phi}{\partial x}\right)_{i-\frac{1}{2},j,k}\right)\frac{1}{\Delta x}$$

Then, each sub-term is again discretized with a central scheme:

$$\left(\frac{\sigma_{i+1,j,k} + \sigma_{i,j,k}}{2} \; \frac{\Phi_{i+1,j,k} - \Phi_{i,j,k}}{\Delta x} - \frac{\sigma_{i,j,k} + \sigma_{i-1,j,k}}{2} \; \frac{\Phi_{i,j,k} - \Phi_{i-1,j,k}}{\Delta x}\right)\frac{1}{\Delta x} \tag{B}$$

The `y` and `z` terms of the Eq. A are treated analogously. The resulting equation is solved for $\Phi_{i,j,k}$ through a standard relaxation procedure.

The electric field is computed through the gradient of the electrostatic potential with a central difference scheme:

$$E \simeq -\left(\frac{\Phi_{i+1,j,k} - \Phi_{i-1,j,k}}{2\ \Delta x}, \frac{\Phi_{i,j+1,k} - \Phi_{i,j-1,k}}{2\ \Delta y}, \frac{\Phi_{i,j,k+1} - \Phi_{i,j,k-1}}{2\ \Delta z}\right) \tag{C}$$

where the derivative of Φ is approximated as:

$$\frac{\partial \Phi}{\partial x} \simeq \frac{\Phi_{i+1,j,k} - \Phi_{i-1,j,k}}{2\ \Delta x}$$

The bioheat equation is written:

$$\rho\ C_p\ \frac{\partial T}{\partial t} = \frac{\partial}{\partial x}\left(k\ \frac{\partial T}{\partial x}\right) + \frac{\partial}{\partial y}\left(k\ \frac{\partial T}{\partial y}\right) + \frac{\partial}{\partial z}\left(k\ \frac{\partial T}{\partial z}\right) - \rho_b\ \omega_b\ C_b\ (T - T_a) + \sigma\ |\nabla \Phi|^2 + q^m \tag{D}$$

The first three terms from the right side of the Eq. D are similar to those in Eq. A, therefore having identical treatment, for instance, the first term becomes:

$$\left(\frac{k_{i+1,j,k}+k_{i,j,k}}{2}\ \frac{T^n_{i+1,j,k}-T^n_{i,j,k}}{\Delta x}-\frac{k_{i,j,k}+k_{i-1,j,k}}{2}\ \frac{T^n_{i,j,k}-T^n_{i-1,j,k}}{\Delta x}\right)\ \frac{1}{\Delta x}$$

the term $\sigma\ |\nabla\Phi|^2$ in the Eq. D is approximated in a similar way to that in Eq. C. The final discretization reads:

$$\rho\ C_p\ \frac{T^{n+1}_{i,j,k}-T^n_{i,j,k}}{\Delta t}=\left(\frac{k_{i+1,j,k}+k_{i,j,k}}{2}\ \frac{T^n_{i+1,j,k}-T^n_{i,j,k}}{\Delta x}-\frac{k_{i,j,k}+k_{i-1,j,k}}{2}\ \frac{T^n_{i,j,k}-T^n_{i-1,j,k}}{\Delta x}\right)\frac{1}{\Delta x}...$$
$$-\rho_b\ \omega_b\ C_b\ (T^n-T_a)+\sigma_{i,j,k}\ \left(\left(\frac{\Phi_{i+1,j,k}-\Phi_{i-1,j,k}}{2\Delta x}\right)^2+...\right)+q^m$$

The previous finite difference scheme was solved for $(T^{n+1}_{i,j,k})$ with an standard explicit method.

To compute the electric current (Eq. 6), the domain is partitioned by half, leaving one electrode in each half-domain. The integral is computed across the surface of one of the halves. For example, to calculate the outgoing flow through one of the half-domain surfaces (when x = ii/2), the discretization is computed as:

$$\sum_{j=0}^{jj-1}\sum_{k=0}^{kk-1} J_x(ii/2,j,k)\cdot(1,0,0)\,\Delta y\,\Delta z \tag{E}$$

Analogously, to calculate the outgoing flow when $x=0$:

$$\sum_{j=0}^{jj-1}\sum_{k=0}^{kk-1} J_x(0,j,k)\cdot(-1,0,0)\,\Delta y\,\Delta z \tag{F}$$

These calculations are similar for `y` and `z` axis.

Boundary Conditions

For every electrode node at the upper surface ($k=0$), Eq. 8 was discretized using a first order scheme:

$$-\kappa\ \frac{\partial T}{\partial z}=h\ \left(T-T_r\right)$$
$$-\kappa\frac{T^{n+1}_{i,j,0}-T^{n+1}_{i,j,1}}{\Delta z}\approx h\ \left(T^{n+1}_{i,j,0}-T_r\right)$$
$$T^{n+1}_{i,j,0}-T^{n+1}_{i,j,1}\approx\frac{-h\ \Delta z}{\kappa}\ \left(T^{n+1}_{i,j,0}-T_r\right)$$

If $r=\frac{-h\ \Delta z}{\kappa}$, then

$$T^{n+1}_{i,j,0}-r\ T^{n+1}_{i,j,0}\approx -r\ T_r+T^{n+1}_{i,j,1}$$
$$T^{n+1}_{i,j,0}\ (1-r)\approx -r\ T_r+T^{n+1}_{i,j,1}$$
$$T^{n+1}_{i,j,0}\approx\frac{-r\ T_r+T^{n+1}_{i,j,1}}{(1-r)}$$

The other boundaries are six rectangular surfaces exposed to the air (due to the box-shape domain). Temperature and electric potential are subject to the Neumann boundary conditions according to Eq. 9 and

Eq. 10. Particularly, for the left surface, i.e. an yz plane, the boundary conditions are:

$$-\kappa \left(\frac{\partial T}{\partial x}, \frac{\partial T}{\partial y}, \frac{\partial T}{\partial z} \right) \cdot (1,0,0) = 0 \tag{G}$$

Recalling that $\kappa \neq 0$ and using a first order scheme in the approximation of the derivatives of Eq. G, the following results: $T_{0,j,k} = T_{1,j,k}$. The remaining boundaries are computed analogously.

Compilation and running script options

NOTE: All files *.cpp and *.h used to generate the simulation results can be accessed at `https://github.com/LSC-UBA/OpenEP/tree/master/src`

`run.sh` **options**	**Description**
`-d` <`simulation-directory`>	Define the simulation directory. Default: `simulation-i`, where i is the next number regarding to the previous simulation directory.
`-n` <`number-of-threads`>	Define the number of threads that will be used by the simulation. Default: number of logical cores of the computer node.
`-c` <`compilation-option`>	Define the compilation option (see Table S.2). Default: `FAST_OMP`.

Table S.1: Compilation and execution commands: `run.sh` bash script.

**Compilation option**	**Description**
`STD`	Standard compilation.
`O3`	Compilation with `O3` optimizations.
`FAST`	Compilation with `Ofast` optimizations.
`DBG`	Compilation with `debug` mode.
`OMP`	Compilation with `OpenMP`.
`O3_OMP`	Compilation with `O3` optimizations and `OpenMP`.
`FAST_OMP`	Compilation with `Ofast` optimizations and `OpenMP`.

Table S.2: Compilation options. In all cases warnings (`Wall` and `Wextra`) are displayed.

	Description	Variable name	Value Example 1	Value Example 2	Value Example 3	Units
**Electrodes**	Electrode length	electrode_length	7.00E-03		19.00E-03	m
	Electrode width	electrode_width	1.50E-03	1.00E-03	7.00E-03	m
	Electrode thickness	electrode_thickness	1.50E-03	1.00E-03	1.00E-03	m
	Space between anode-cathode	gap_anode_cathode	8.00E-03	1.00E-02	8.00E-03	m
	Type of electrode	electrode_type	"needles"	"needles"	"plates"	
	Space between anode-anode and cathode-cathode (only with needles)	gap_elect_elect	5.00E-03	5.00E-03	5.00E-03	m
	No. of electrodes (only with needles)	no_electrodes	1	3	2	
**Domain parameters**	Maximum size of the x-axis	x_max	3.30E-02	4.00E-02	2.00E-02	m
	Maximum size of the y-axis	y_max	3.30E-02	5.00E-02	1.5E-02	m
	Maximum size of the z-axis	z_max	1.10E-02	2.00E-02	2.5E-02	m
**Treatment parameters**	Voltage to distance ratio	volt_to_dist	25000	150000	25000	V / m
	Frequency	freq	1	1	1	Hz
	On time	on_pulse_time	5.00E-02	1.00E-04	5.00E-02	s
	No. of pulses	nbr_pulses	8	32	8	
**Other treatment parameters, calculated from the above**	Duration of one pulse	total_pulse_time	1. / freq	1. / freq	1. / freq	s
	Duration of the OFF period	off_pulse_time	total_pulse_time - on_pulse_time	total_pulse_time - on_pulse_time	total_pulse_time - on_pulse_time	s
	Simulation total time.	max_time	nbr_pulses * total_pulse_time	nbr_pulses * total_pulse_time	nbr_pulses * total_pulse_time	s
	Maximum voltage between electrodes	max_voltage	volt_to_dist * gap_anode_cathode	volt_to_dist * gap_anode_cathode	volt_to_dist * gap_anode_cathode	V
	Electric potential at the anode	phi_anode	max_voltage	max_voltage	max_voltage	
	Electric potential at the cathode	phi_cathode	0	0	0	
	Electric potential value at initial time	phi_init	0	0	0	
	Electric field value at initial time	ef_init	0	0	0	V / m
**Tissue parameters**	Temperature value at initial time	temperature_init	310.15	298.15	310.15	K
	Thermal conductivity at initial time	k_domain_init	0.512	0.512	0.512	W / (m K)
	Electric conductivity	sigma_domain_init	0.126	3.00E-02	0.504	S / m
	Tissue density	rho	1050	1100	1050	kg / m3
	Heat capacity	cp	3600	3780	3600	J / (kg K)
	Metabolic heat generation	qm	420	2161	420	W / m3
**Blood parameters**	Temperature of the arterial blood	temperature_b	310.15	0	310.15	K
	Density of the blood	rho_b	1060	0	1600	kg / m3
	Heat capacity of the blood	c_b	3600	0	3600	J / (kg K)
	Blood profusion	w_b	0.0044	0	0.0044	1/s
**Electrode parameters**	Electrode density	rho_electrode	1050	1050	1050	kg / m3
	Heat capacity	cp_electrode	490	490	490	J / (kg K)
	Electric conductivity	sigma_electrode_init	1.40E+06	1.40E+06	1.398E+06	S/m
	Thermal conductivity	k_electrode_init	16.3	16.3	16.3	W / (m K)
**Boundary parameters**	Convective heat-transfer coefficient	h	10	25	10	W / (m2 K)
	Adim.	alpha0	0.015	0.015	0.015	
	Air temperature	temp_air	298.15	298.15	298.15	K

Table S.3: Model parameters used in the examples.

Description	Variable name	Example 1	Example 2	Units
Resolution (No. of nodes between anode and cathode)	resolution	30	30	
No. of x axis divisions	ii	120	119	
No. of y axis divisions	jj	120	150	
No. of z axis divisions	kk	63	59	
Space between nodes in x, y and z axes	dx, dy, dz	2.6E-04	3.00E-04	m
Maximum number of sub-iteration at begining	max_sub_it_Phi_init	20000	10000	
Maximum number of sub-iteration during main simulation	max_sub_it_Phi	50	25	
Relaxation	omega	1	1	
Time step during on pulse	dt_on_pulse	on_pulse_time / 500	on_pulse_time / 500	s
Time step during off pulse	dt_off_pulse	on_pulse_time / 500	on_pulse_time / 500	s
Save each ... iterations	save_step	1 / dt_on_pulse	1 / dt_on_pulse	
Log each ... iterations	log_step	500	500	
Save format ("vtk" or "csv")	save_format	"vtk"	"vtk"	

Table S.4: Numerical parameters used in the examples.

How to optimize an EP-based treatment in terms of pulse number in five steps.

1. Compute with the OpenEP simulator the electric field variation in space and time for a given EP-based treatment, such as the time-invariant red curve shown in the Paraview 1 D plot in figure 1 (for the

inset plot, see next episode)

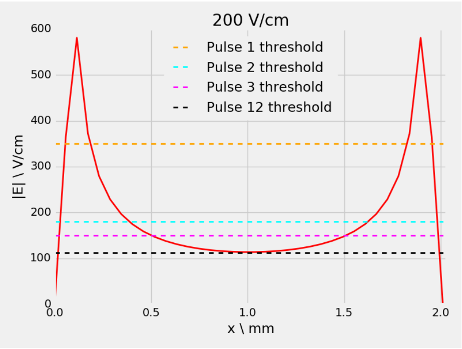

Figure 1: The electric field variation in time (red line) which is time-invariant.

2. Assume an exponential time decreasing function for the threshold variation in time, based on the specified tissue type and experimental measurements from literature[1], such as the blue curve shown in the Paraview 1D plot in figure 2.

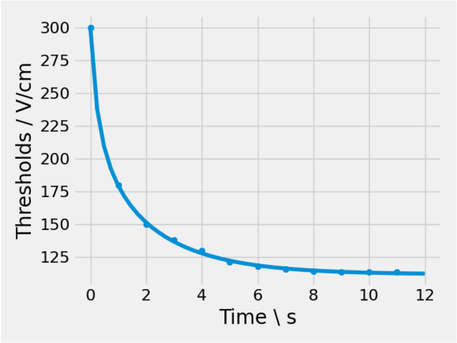

Figure 2: The exponentially time decreasing threshold function assumed.

3. With the threshold values from the blue curve plot from figure 2, draw horizontal cross-sections on the electric field variation red curve plot (dashed color lines), such as in the next Paraview 1D graph in figure 3. Each dashed line determines the electroporated tissue area associated with the pulse number. For instance, the thresholds in the first and twelfth pulses define electroporated tissue occupying 10 percent and 100 percent, respectively, of the distance between electrodes (graph taken from Lujan et al. 2019 E. Acta).

[1] In the first pulse, the threshold function value can be approximated by the highest electric field isoline (close to the electrode) coming from the electric field distribution. This is because, in the first pulse, the measured electroporated area would be tiny (close to zero in the first instants); therefore, the electric field isoline matching this electroporated area will be very high. Usually, for the type of tissue treated, literature offers threshold measurements for the nth. pulse, and seldom for different pulses. **Hint**. A good approximation of the exponential decreasing threshold function would be as follows: taking as an example the figure in item iii), divide the x-axis in the interval [0,1] (due to symmetry) in 12 segments (corresponding to the 12 pulses used in this example). For each segment, from 1 to 12, measure the corresponding electric field. The electric field vs pulse number graph obtained approximates the unknown exponential decreasing threshold function.

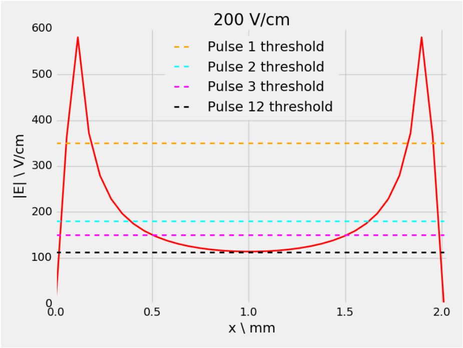

Figure 3: The electric field variation in time (red line) which is time-invariant, and the horizontal cross-sections (dashed color lines) determining electroporated tissue areas for different pulses.

4. Draw the electroporated tissue area variation in time (electroporated tissue trajectory) from the previous figure 3, such as in the next Paraview plot in figure 4 (here, since damage is not considered, treated tissue coincides with electroporated tissue).

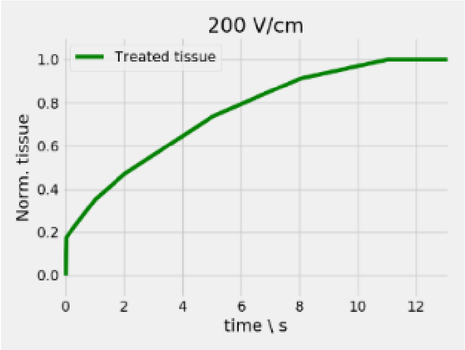

Figure 4: Electroporated tissue area variation in time

5. Having the electroporated tissue trajectory, it is possible to determine the pulse dosage maximizing electroporated tissue with minimum damage (if the damage is not included, as in the green curve, the optimum pulse dosage (pulse number) is obtained as the first pulse in which the maximum difference between EA (n+1) − EA(n) is smaller than a prescribed small value). It is optimal because further pulsing will not increase the electroporated tissue area but may introduce damage.
